# Contributing and mitigating factors of brain drain among healthcare workers from cardiac care facilities in Nepal - A qualitative case study

**DOI:** 10.1371/journal.pgph.0004260

**Published:** 2025-03-25

**Authors:** Niharika Jha, Rashmi Maharjan, Swornim Bajracharya, Soniya Shrestha, Bobby Thapa, Pranil Man Singh Pradhan, Sushmita Mali, Biraj Man Karmacharya, Archana Shrestha

**Affiliations:** 1 Department of Epidemiology and Biostatistics, University of Memphis, Memphis, Tennessee, United States of America; 2 Department of Community Programs, Dhulikhel Hospital, Dhulikhel, Nepal; 3 Department of Epidemiology, University of Washington, Seattle, Washington, United States of America; 4 Department of Nursing and Midwifery, Kathmandu University School of Medical Sciences, Dhulikhel, Nepal; 5 Institute for Implementation Science and Health, Kathmandu, Nepal; 6 School of Public Health, Patan Academy of Health Sciences, Lalitpur, Nepal; 7 Department of Nursing, Tribhuvan University, Institute of Medicine, Nepalgunj Nursing Campus, Banke, Nepal; 8 Department of Community Medicine, Maharajgunj Medical Campus, Institute of Medicine, Tribhuvan University, Kathmandu, Nepal; 9 Department of Public Health, Kathmandu University School of Medical Sciences, Dhulikhel, Nepal; 10 Department of Chronic Disease Epidemiology, Yale School of Public Health, New Haven, United States of America; PLOS: Public Library of Science, UNITED STATES OF AMERICA

## Abstract

The migration of health experts seeking better opportunities, both nationally and internationally, is a growing concern due to its impact on health systems, particularly in developing countries. The provision of cardiovascular and other specialized medical care requires a skilled workforce, yet the factors driving brain drain among cardiac care health workers remain underexplored. This study assessed the factors influencing the brain drain of cardiac healthcare workers from the perspective of the health policymakers and health care workers in Nepal. We conducted a cross-sectional qualitative study among 32 key informants selected purposely working at the policy level, tertiary cardiac care hospitals, universities, cardiac care civil societies, and medical, nursing, and public health professional councils in Nepal. We interviewed the participants using a standardized key informant interview guide with open-ended questions probing for in-depth information in the Nepali language. The interviews were audio-recorded, transcribed, coded, and analyzed using the thematic method. We used the inductive method of data analysis and manually developed codes and themes from the transcripts ensuring a robust analysis of the migration factors impacting cardiac healthcare workers. Key findings revealed several contributing factors to brain drain, including better job opportunities, higher pay scales, and improved working environments in developed countries. Push factors such as young age, family attitudes toward migration, and low levels of patriotism among healthcare professionals were also identified. Addressing these issues requires targeted retention strategies, including creating opportunities within the country, fostering collaboration between policymakers and stakeholders, and enhancing working conditions in Nepal’s healthcare sector. The global public health implications of brain drain are significant, underscoring the need for sustainable solutions to strengthen healthcare systems and promote health equity. Developing and implementing policies that mitigate brain drain will be crucial to retaining skilled cardiac healthcare workers and ensuring the delivery of quality care in Nepal.

## Introduction

An effective healthcare system is dependent on the right number, distribution, and mix of health workers [1]. However, the health workforce is unevenly distributed worldwide with a shortage of 15 million health workers [2,3]. In many low- and middle-income countries, achievement of health development goals is hindered by acute shortages and inequitable distribution of skilled health workforce. These countries face a human resource (HR) crisis due to three major factors: 1. unavailability of qualified health workers; 2. uneven distribution due to low recruitment and poor retention issues; and 3. insufficient productivity and quality of care they provide [4]. The migration of medical professionals from developing countries to developed nations is a major concern, worsening the already depleted healthcare resources in poor countries and widening the health inequity gap worldwide [5]. Medical brain drain is a major factor contributing to insufficient medical personnel, whose consequences are affecting not only health systems but also economic development and national security [6].

Nepal has 21 medical colleges[7] and annually produces about 2000 medical doctors [8], 7610 nurses (nurses, midwives)[9], and 6540 paramedics (HA 1880, lab workers 2520, pharmacy 1050, public health 640, radiographer 450) [10]. Despite the mass production of skilled healthcare workers, their consumption is very scarce in the national labor market [11]. Currently, there are a total of 1154 specialists and 85,042 general nurses registered in Nepal [12], but there still is a shortage of nurses in the public sector, local hospitals, and primary health centers (PHCs) [13]. The patient-doctor ratio in Nepal is 1,721:1 and the patient-nurses ratio is 500:1 [14]. This is way lower than the World Health Organization (WHO) recommendation of a 1000:1 patient-doctor ratio and a 1000:2.3 patient-nurse ratio [13]. Like most developing nations, doctors are geographically maldistributed in Nepal. The Kathmandu Valley has one doctor for 850 people but in rural areas, there is one doctor for every 150,000 people [15,16]. The doctor-population density in Kathmandu is estimated to be about 40 times that in rural Nepal [16]. Nepal even lacks accurate data on how many doctors, nurses, and other health care personnel are leaving the country each year.

Given the substantial time and resources invested in educating and developing skilled health workers in developing countries like Nepal, it is crucial to understand the factors that affect the migration of this health workforce jeopardizing the investment of the health system. Key professional reasons influencing medical professionals to emigrate include poor remuneration, bad working conditions, an oppressive political climate, lack of opportunity, high unemployment in health labor markets, poor facilities, limited career structures, and poor intellectual stimulation [17]. In addition, personal reasons such as lack of security, the threat of violence, and the wish to provide a good education for their children also contribute to their migration [5].

Cardiovascular Disease (CVD) is a major public health issue in Nepal. There were an estimated 1,214,607 cases of CVD, in 2019, which resulted in 46,501 deaths in Nepal [18]. However, none of the studies have explored migration among cardiac healthcare workers in Nepal. Hence, in this qualitative paper, we explored the perception of policymakers and cardiovascular healthcare providers on brain drain in Nepal and its influencing factors. This research enables us to unveil the perspectives and experiences of healthcare workers themselves, as well as stakeholders such as policymakers and medical university administrators. Although this study focuses on the migration patterns of healthcare workers, “brain drain” is considered a potential consequence of migration. Our primary aim is to explore the factors contributing to and mitigating the migration of cardiac healthcare workers that may lead to brain drain over time. Hence, it provides a rich understanding of the multiple factors influencing brain drain.

## Methods and materials

### Ethics statement

This study was approved by the ethical review board of the Nepal Health Research Council (NHRC) (Reg. no.176/2018). All the participants provided written informed consent.

### Study design

This was an exploratory cross-sectional qualitative study. We conducted desk reviews of existing human resource-related reports and policies in Nepal and key informant interviews with representatives of policymakers, professional council organizations advocating for cardiac patients, medical universities, and cardiac health care professionals at the central level.

### Desk review

We reviewed existing policies, annual reports, acts and regulations, and national and international journals to explore the existing status of brain drain in Nepal (Table 1). For those documents that were not available online, we gathered necessary information directly by visiting the concerned centers, and offices. We stored all secondary literature on an online storage named “Secondary Data Sources”. We also extracted various documents from health professional councils, ministries (Health and Education), and annual reports from tertiary cardiac care centers: Shahid Gangalal National Heart Centre (SGNHC), Manmohan Cardiothoracic Vascular and Transplant Center (MCVTC), Bir Hospital, and B.P. Koirala Institute of Health Sciences (BPKIHS). For that, first, we visited the places with letters provided by Dhulikhel Hospital Kathmandu University Hospital (DH-KUH). We got approval from the administration section of each organization and received the information. We conducted a Desk Review from March 2019 to June 2019.

**Table 1:** HRH-related acts/policies in Nepal.

Acts/Policies	Description
Public Health Service Act 2018 ^a^	The Government of Nepal shall prepare and enforce the policy and standards relating to the development, distribution, and use of human resources of the health sector. While prescribing standards referred to in subsection (1), such standards shall be prescribed based on estimation of human resources, technology, and equipment by analyzing and mapping long-term, mid-term and immediate requirements for the development, management, and use of the human resource.
Nepal Health Sector Strategy 2015-2020 ^b^	Given the current context of a critical shortage of health workers particularly in rural areas, retention of available health workers also remains a priority area for the next five years. Develop effective mechanisms for efficient recruitment and distribution of health workers in remote areas, including incentive mechanisms.
Rural staff support program, Nepal, 2007–2015 ^c^	The central component of the rural staff support program was recruiting one- or two-family practice doctors per program hospital. These physicians are post-graduate doctors trained in medical universities in Nepal – to provide primary care as well as basic surgical, orthopedic, and obstetric services.
Human Resources for Health (HRH) Strategic Plan 2011-2015 ^d^	Five key HRH problem areas and issues were identified and discussed: Shortages of HRH as a result of imbalances between supply and demand, maldistribution of staff, especially in remote and rural areas, poor staff performance, including productivity, quality, and availability, fragmented approaches to human resource planning, management and development, HRH Financing
Health Worker and Health Institution Security Act 2010 ^e^	If any person commits or attempts to commit any act against any health worker or any health organization in contravention of Section 3, such a health worker or health organization may make a request to the local administration for security. If a request for security is made under sub-section (1), the local administration shall have to arrange for security immediately. If, given any place and situation, obstruction in the services to be provided by any health organization is likely to occur, and permanent arrangement of security is necessary, and the committee writes for such security, the Government of Nepal may arrange for security in such a health organization permanently.

^a^The Public Health Service Act, 2075 (2018) https://www.lawcommission.gov.np/en/wp-content/uploads/2019/07/The-Public-Health-Service-Act-2075-2018.pdf

^b^NEPAL HEALTH SECTOR STRATEGY 2015 - 2020 https://nepal.unfpa.org/sites/default/files/pub-pdf/NHSS-English-Book-final-4-21-2016.pdf

^c^A staff support programme for rural hospitals in Nepal 2007- 2015 (https://www.ncbi.nlm.nih.gov/pmc/articles/PMC4709798/pdf/BLT.15.153619.pdf)

^d^Human Resources for Health Strategic Plan 2011-2015 https://www.who.int/workforcealliance/countries/Nepal_HRHStrategicPlan_finaldraft.pdf?ua=1

^e^Security of the Health Workers and Health Organizations Act, 2066 (2010) http://nepalpolicynet.com/images/documents/publichealth/acts/security-of-the-health-workers-and-health-organizations-act-2066-2009-e.pdf

### Key informant interviews

To fill the gap in the information following the desk review we conducted key informant interviews (KII) with national-level policymakers, cardiac healthcare workers, deans of different universities, and representatives from different civil societies from 10th June 2019 to 10th September 2019. We included participants only from federal-level policymakers and cardiac healthcare providers, as they often have a comprehensive understanding of the resources available and the challenges for cardiac care in the nation. Interviewing them provides valuable insights into existing policies related to healthcare workforce retention, migration, and strategies for mitigating brain drain. We developed a list of relevant agencies to identify key informants (KIs) and purposely selected 32 informants using our professional networks. We developed a semi-structured questionnaire and used it as a KII guide. Our interview guide included questions like (1) How do you define “brain drain “ in the context of cardiac healthcare workers in Nepal? (2) From your perspective, what are the main reasons why cardiac healthcare workers choose to migrate from Nepal? (3) What strategies or interventions could effectively mitigate brain drain among cardiac healthcare workers in Nepal? (4) How do existing policies and regulations affect healthcare workers’ decisions to stay or leave Nepal? (5) What are the possible impacts of this brain drain?

We pre-tested the KII tool at DH-KUH and we did not include those participants in the final analysis. At first, we approached each KI with the questionnaire and set an appointment. On a day convenient to our participants, we reached their offices and interviewed the participants using a standardized key informant interview guide. Following informed consent from the participant, we began our interview with an introduction section that collected demographic data of the interviewee and then continued the interview in the Nepali language. We conducted all the interviews privately and did not record any video or picture identifying the key informant. We audio-recorded all the interviews, and a note taker took notes, no interview exceeded an hour. Principal author (NJ) and a co-author (SS) were mainly involved in all the data collection. This study has a 100% participation rate.

### Data management and analysis

**Fig 1:**
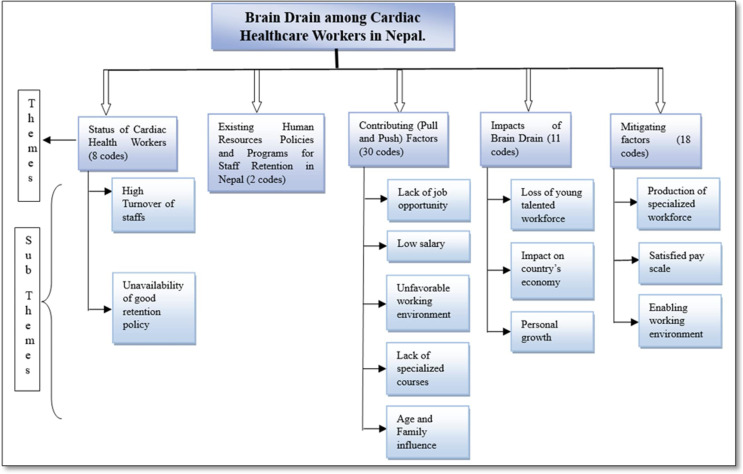
Code tree of the study.

We transcribed all the recordings in the Nepali language verbatim using Express Scribe transcription software and structured protocol for transcription. The research team verified the transcript against audio recordings to ensure completeness and accuracy. We used a thematic analysis method for data analysis. First, we read all the interviews several times to understand the raw data and generated codes by transforming information into meaningful categories. We developed a codebook on which we (NJ and RM) separately coded all the interview transcripts. We used the inductive coding method, and two coders discussed discrepancies as necessary. After comparing all the interview transcripts and proposed codes, a third researcher (SB) generated an intercoder agreement of 83.45% in this study. We conducted the interviews till we observed data saturation. We generated 69 codes from all the transcripts and broadly categorized them into 6 themes (S1 Data). All the hard copies of the transcripts are secured with limited access to concerned members only. This study used COREQ (COnsolidated criteria for REporting Qualitative research)[19,20] guidelines for reporting and presenting the findings. Fig 1 illustrates a coding tree based on the findings.

## Results

### Characteristics of the participants

Out of 32 participants, 44% were female and the majority (46.9%) were cardiac care providers. The mean age of the key informants was 46 (± 8.6) years and the majority of them (84%) had completed master’s degrees in their respective areas. Table 2 presents the characteristics of the study participants.

**Table 2:** Characteristics of the study participants (n = 32).

Demography	Frequency (%)
**Gender**	
Male	18 (56)
Female	14 (44)
**Qualification**	
Master’s and above	27 (84.4)
Bachelors	3 (9.4)
Intermediate level	2 (6.3)
**Representative of**	
Policymakers	9 (28.1)
Professional council	3 (9.4)
Civil societies	2 (6.3)
Medical Universities	3 (9.4)
Health service providers	15 (46.9)
**Age (years) mean (SD)**	46 years (8.6)
**Years of experience mean (SD)**	20 years (8.8)

***Note:*** *SD= Standard Deviation*

We developed 69 codes from all the transcripts. Later, we integrated findings from the desk review findings with KII codes and generated 6 broad themes: (A) the status of cardiac healthcare workers in Nepal, (B) existing human resources policies and programs for staff retention, (C.1) push factors contributing to brain drain, (C.2) pull factors contributing to brain drain, (D) effects of brain drain, and (E) Factors mitigating to brain drain.

We described all the broad themes and sub-themes with the supporting statements of the study participants for a comprehensive understanding of the contributing and mitigation factors of brain drain among Cardiac healthcare workers in Nepal.

#### Status of cardiac healthcare workers in Nepal.

This theme explains the conditions, and challenges in the current healthcare system within the context of Nepal. The cardiologist-patient ratio is 0.0057 per thousand population; the nurse-patient ratio is 2.85 per thousand population in Nepal. Only 48.2% of sanctioned medical staff posts and 77.96% of sanctioned nursing staff posts were filled in the public sector. The information for the private sector is not available.

Key informant interviews (KIIs) revealed that nursing and medical faculties have been more heavily impacted by brain drain compared to cardiovascular specialists. However, hospitals in Nepal continue to experience high staff turnover, creating an ongoing need for substantial investment in training new cardiac specialists.

Participants highlighted that Nepal produces a competent healthcare workforce, but many doctors, including cardiologists, leave for opportunities abroad after completing their education. A participant expressed concern about this high turnover:

*“Trained staff were present here first. Then they leave, and new staff are recruited. Again, we have to train the new staff. So, I must say turnover is present in a huge amount here. Last time when we had open vacancies, we had 100 people on the waiting list, but now the list is almost empty. So, we can say that 100 staff were replaced here. Even permanent staff left their jobs.” -* A staff nurse

Another participant shared a similar sentiment, underscoring the impact of brain drain on highly skilled professionals:

*“We are losing educated manpower due to brain drain in Nepal. They definitely have a place in Nepal to work, right? But… I met two doctors who were working in Japan. One has returned here, but another is still deciding whether to come back or not. A cardiologist of Nepal, working in Japan…”* - A representative from the Nepal Heart Foundation (NHF)

#### Existing human resources policies and programs for staff retention in Nepal.

This theme explores the current human resources policies and programs implemented in Nepal aimed at retaining staff within various health sectors, highlighting strategies designed to address workforce turnover and promote employee retention across the country.

The Government of Nepal, in collaboration with the Nick Simons Institute, initiated a rural staff support program in 2007 to improve the retention of healthcare workers in remote hospitals. The program offers post-graduate academic support, financial incentives, career advancement opportunities, allowances, enhanced diagnostic facilities, and security measures for rural health workers.

Despite these efforts, key informant interview (KII) participants expressed skepticism about the effectiveness of these policies in retaining healthcare professionals.

*“The government says, ‘don’t go abroad, don’t go...’ but we do not have any policies to retain them here.”* - A representative from the Ministry of Health and Population (MoHP)

This reflects a broader perception that current retention strategies are insufficient to address the pull factors drawing health workers, particularly skilled professionals, to international opportunities. The insights suggest that without more comprehensive policy measures, brain drain and workforce shortages in rural hospitals will continue to challenge Nepal’s healthcare system.

#### Factors contributing to brain drain.

We summarized the contributing (pushing and pulling) factors to brain drain in Table 3. The most reported pushing factors are inadequate workplace safety and security, inadequate facilities to provide health services, and pulling factors are better job opportunities, pay scale, and opportunities for higher education in developed countries.

**Table 3:** Contributing and mitigating factors of brain drain among cardiac healthcare workers in Nepal.

Pushing factors	Pulling factors	Mitigating factors
Inadequate workplace safety and security Poor working facilities/opportunities Lacking specialized health-related courses Low recruitment of specialized health personnel Lack of a sustainable retention plan Low pay scale Personal/family influence Young Age	Job opportunities Large payment in abroad Better education opportunities Good working environment and facilities	Create suitable posts for health workers. Develop a sustainable retention plan (good salary, facilities, and opportunities) Initiation of specialized health programs in universities

***Pushing factors:*** This theme delves into the specific factors within Nepal that prompt individuals to leave the country in pursuit of opportunities elsewhere. Below, we explained the key push factors contributing to brain drain identified from the data.

I Inadequate workplace safety and security:

A 2013 study on ‘Perception of security by the health workforce at workplace in Nepal’ reported that 23% of the health workers felt a secure workplace environment [11]. Only 30% of the health workers who got any injury at the workplace have received compensation [11].

Key informants highlighted that healthcare workers in Nepal face both physical and mental safety challenges. Doctors and nurses are often held accountable by the public for unfavorable patient outcomes, making them targets of blame and hostility. This environment creates significant risks for healthcare professionals, impacting their well-being and job satisfaction.

*“The medical profession is attacked by local people in such a way nowadays, no? In that way, doctors do not want to stay here in Nepal. Because of this violence also, many of them are leaving the country.” – a consultant Cardiologist.*

*“Our country has many big jobs like improving the economy, improving the political stability, and also improving the social and physical security of health workers to control brain drain in Nepal.” – a cardiothoracic surgeon.*

II Inadequate facilities to provide health services:

The infrastructure, resources, and personnel within Nepal’s healthcare system are insufficient to meet the population’s growing needs, leading to compromised delivery and quality of care. One participant highlighted that health workers in Nepal face significant difficulties due to the scarcity of resources, making it challenging to provide optimal care. The lack of essential tools and facilities creates frustration and dissatisfaction among staff, diminishing their ability to perform their duties effectively.

*“Doctors need an ECG machine, X-ray machine, a place to work in the lab, and a place to stay, right? They also need internet service to update themselves. One responsible organization or the government should make all these facilities available so they can work efficiently in their respective areas.” — a representative from a medical university.*

This inadequacy is reflected in various ways, including limited access to essential medical equipment, under-resourced facilities, and a lack of supportive infrastructure for health workers. These challenges negatively impact both patient outcomes and health worker morale.

III Low pay scale:

In Nepal, there is a significant imbalance between the production of healthcare professionals and their actual deployment and utilization within the healthcare system.

Key informants emphasized that the shortage of manpower in many health facilities places a heavy workload on cardiologists and other doctors. This excessive workload leads to heightened stress and professional dissatisfaction, particularly as they feel undercompensated for the long hours they work.

*“Working facilities are lacking in Nepal. Specialists or doctors have more workload. Manpower is less, and workload is more. They are stressed due to work and do not receive remuneration accordingly.” — a consultant cardiologist.*

This statement highlights the dual challenges faced by healthcare professionals: insufficient staffing and inadequate compensation. These factors contribute not only to burnout but also to a growing sense of dissatisfaction, which can affect the quality of care and increase the risk of brain drain from the healthcare system.

IV Lack of specialized health-related courses in Nepal:

The scarcity of specialized health-related courses in Nepal highlights a critical gap in the country’s healthcare education system. Participants expressed that very few medical universities and institutions offer advanced or specialized medical programs, limiting opportunities for students to pursue higher education within the country. As a result, many students, after completing their bachelor’s degrees, are forced to seek opportunities abroad to continue their education and achieve their career goals.

*“Specialized master’s level courses related to bachelor-level degrees are not available. We don’t have enough institutions or universities to absorb all graduates. Very few universities offer specialized courses, leaving students with limited options, so they go abroad for higher education.”* — a representative from the health professional council.

This gap in educational infrastructure not only contributes to the migration of aspiring health professionals but also exacerbates the brain drain within Nepal’s healthcare sector. Without the availability of advanced training and specialization programs, the country struggles to retain talent, impacting the long-term development of the healthcare workforce.

V Personal/family influence

The pursuit of social recognition and financial security becomes a key motivator, contributing to the brain drain of healthcare professionals and students from Nepal.

*“The opinion of our society and family is also one reason. Our society thinks that if your children are not abroad, your family status is quite poor. So, to show your prestige in society, families influence their children to go abroad for education or career development.”* — a dean of a medical school.

Economic factors within the family also contribute to migration decisions. When families face financial difficulties and the local economy offers limited opportunities, migration becomes a practical solution for improving their quality of life. In such cases, family members may actively support migration in the hope of securing better financial prospects and stability.

VI Age:

The age group of 20 to 30 years is especially inquisitive and inclined toward exploration. Individuals at this stage of life are eager to experience new cultures, places, and lifestyles, which fuels their desire to migrate.

*“Brain drain also depends upon the age of an individual. The age between twenty to thirty is a vulnerable age, and that age is very inquisitive. As children are curious about learning, likewise at this age people are curious about careers. They want to explore the outside world. They have seen it on television and want to experience international flights, roads, environments, and food. They are curious about New York, the London Tower, etc. This is about the perception of an individual. We cannot force them to stay in their own country and develop their place.”* — a dean of a medical school.

This insight of the participant reflects how age-specific curiosity, exposure to global influences, and career aspirations contribute to brain drain. Younger professionals’ drive to explore the world and seek better opportunities is a natural part of their developmental stage, making them more inclined to migrate, despite efforts to retain them locally.

***Pulling factors:*** This theme explains the external factors outside of Nepal that attract skilled professionals to migrate abroad which contribute to the pull factors exacerbating brain drain from Nepal. We detailed these factors below.

I Better job opportunities abroad:

A recurring theme from key informant interviews (KIIs) was the disappointment among cardiac healthcare workers over the disparity between their responsibilities and compensation. Many participants expressed that inadequate pay, combined with demanding workloads, fuels thoughts of migration and limits the incentive to remain in Nepal’s healthcare system.

*“After completing MBBS, doctors receive a monthly salary of twenty thousand rupees (around 200 USD) in Nepal. Meanwhile, their friends from other fields earn far more abroad. So how do they stay satisfied with twenty thousand? They won’t be satisfied. That’s why they go abroad—they feel they have no choice. Either we need to increase doctors’ salaries or reduce their working hours.”* — a dean of a medical school.

Beyond salaries, healthcare workers are also drawn to opportunities abroad that offer better working conditions, including advanced medical technology, modern facilities, and resources to provide high-quality care.

II Opportunity for higher education:

Notably, the desire for quality training, rather than inadequate qualifications, is a primary reason for migration. Cardiac healthcare workers are drawn to environments where they can collaborate with multidisciplinary teams, participate in clinical trials, and engage in innovative research—opportunities that are essential for career progression and personal growth but are limited in Nepal.

Key informants also emphasized that brain drain is multifactorial, driven not only by educational constraints but also by individual choices, family expectations, and the influence of the country’s economic and professional landscape. Participants shared that only a small percentage of medical graduates have the opportunity to pursue advanced degrees in Nepal, further compounding the issue.

*“After completing MBBS, only ten percent of them get MDs here in Nepal. Yeah, we have only ten percent of MDs. That means the remaining ninety percent have no choices, right.”* — a cardiologist.

III Large payment in abroad

The higher wages abroad not only allow healthcare workers to better support themselves but also provide more opportunities to care for their families and plan for the future. This financial security becomes a major incentive for migration, especially when compared to the limited economic opportunities available at home.

*“People in other countries work based on hours. The rate is $30 per hour, and they work a minimum of 8 hours, which is $240 a day—far more than the salary for one month in Nepal. So, they choose to work abroad rather than in Nepal.”* — a nursing supervisor.

This quote highlights how the stark difference in compensation structures makes working abroad an attractive option. Without addressing these financial disparities, retaining healthcare professionals in Nepal will remain a significant challenge.

IV Better facilities:

Developed countries typically offer a much higher standard of living compared to Nepal, encompassing access to superior healthcare, education, infrastructure, and public services.

Participants in our study expressed that the excitement of a more comfortable and luxurious lifestyle motivates many to migrate. The perception of better facilities and opportunities for a higher quality of life overseas drives healthcare professionals to seek a future in more developed nations.

*“Another reason is quality of life. Everything we need comes from abroad. That is why people migrate to other countries. Abroad, people receive better education from childhood. The roads, transport, vehicles, and traffic—everything is better than here. So, I think quality of life is one of the major reasons for brain drain.”* — a nursing supervisor.

#### Impact of brain drain on the country.

This theme explores the consequences of brain drain on Nepal, particularly how the migration of skilled professionals adversely affects the development of the country’s healthcare system. While brain drain can have detrimental effects on the nation, it often leads to personal growth and development for the individuals involved.

Some participants noted the potential benefits of brain drain, emphasizing that exposure to different work environments, cultures, and perspectives can broaden one’s horizons and enhance skill sets. This personal growth can foster the acquisition of new technologies and advanced knowledge, which could ultimately benefit Nepal if these individuals choose to return. Additionally, working abroad can contribute to the prosperity of their families.

*“We should not stop them if they want to leave. Yes, it is not right for the country. Health workers produced by Nepal going abroad for work is not good for the country, but it is good for the individual and beneficial for their families.”* — a representative from the Ministry of Health and Population (MoHP).

However, the negative consequences of brain drain for the country cannot be overlooked. The departure of skilled workers creates significant shortages in critical sectors, particularly in cardiac care.

Most participants viewed brain drain as a loss for the nation, leading to a depletion of skilled manpower and a talented workforce, which, in turn, impacts the country’s economic stability. The loss of skilled health workers hampers productivity and restricts Nepal’s ability to diversify its economy and create new job opportunities.

*“We are losing many young generations due to brain drain. Talented people, skilled individuals are leaving the country. We face a tremendous loss due to brain drain.”* — a chief nursing supervisor.

In summary, while brain drain may offer personal advantages to individuals and their families, it poses significant challenges for Nepal’s healthcare system and overall economic development. The dual nature of brain drain underscores the need for strategies to retain skilled professionals while also recognizing the individual’s pursuit of better opportunities.

#### Mitigating factors to brain drain.

This theme identifies and analyzes the measures and strategies to mitigate brain drain in Nepal. Key informants highlighted several critical factors, including the development of retention plans, the creation of suitable positions for health workers, and the initiation of specialized health courses within the country.

I Develop a sustainable retention plan

Our findings from key informant interviews suggest that the government of Nepal should prioritize improving the salary structure of healthcare workers, ensuring that compensation is both satisfying and competitive with international standards. Regular salary reviews and adjustments to reflect market trends and the cost of living are essential. Additionally, the government should offer benefits such as healthcare coverage, retirement plans, professional development opportunities, and performance-based bonuses to attract and retain talent.

*“The first reason is salary, isn’t it? If a person does not get a good place to survive here, how can they stay? So, first, the government must provide them with a good salary to keep them satisfied.”* — a senior staff nurse.

Furthermore, it is crucial for the health workforce to have access to growth opportunities and to foster a sense of commitment to working in their home country. Universities can play a pivotal role by emphasizing social accountability and a community-oriented approach during students’ training.

According to key informants, while brain drain cannot be entirely stopped, it can be controlled by developing opportunities within Nepal to encourage healthcare professionals to remain.

*“If workers develop an emotional attachment to their work, then they are less likely to leave their workplace. Therefore, we should cultivate such a working environment that encourages them to stay here and not leave our country.”* — a cardiologist.

Addressing brain drain in Nepal requires a comprehensive approach that includes competitive compensation, improved working conditions, and opportunities for professional development. By fostering a supportive environment, the country can mitigate the loss of skilled healthcare professionals and encourage them to contribute to the nation’s healthcare system.

II Create suitable posts for health workers

To effectively address brain drain, the government must establish appropriate positions and job opportunities for healthcare personnel. Many health workers find themselves in roles that do not align with their training or interests after completing their studies. This misalignment often forces them to accept lower-ranked jobs, leading to dissatisfaction and contributing to their decision to seek opportunities abroad.

To mitigate this issue, it is crucial for the government to create well-defined roles and responsibilities tailored to the qualifications of health workers. Additionally, the recruitment of new staff can help alleviate the burden on existing healthcare professionals, who often face overwhelming workloads.

*“If they have better opportunities, better posts, and a supportive working environment, then definitely, they will consider returning. For instance, many specialists are coming back to India from America and the UK. If we can create similar opportunities for specialists here, they would likely come back.”* — a representative from the Nepal Health Federation (NHF).

Moreover, addressing the shortage of specialists is vital. Current specialists report experiencing high levels of stress due to inadequate staffing and excessive pressure. While some individuals may still choose to leave despite improvements, it is essential to develop strategies to recruit, train, and retain new healthcare professionals to ensure the sustainability of the healthcare system.

*“When we discuss specialists, we find ourselves overwhelmed with workload. The number of specialists is low, while the pressure is high. Regardless of what is done, people will continue to leave, so we must have strategies to train new personnel and continue providing quality care.”* — a cardiothoracic surgeon.

III Initiation of specialized programs in universities

Establishing specialized programs in universities is essential for providing students with the skills and competencies that are increasingly demanded in the global job market. Our participants highlighted that the Ministry of Health and Population (MoHP), in collaboration with medical universities, should prioritize the development of specialized education programs within Nepal.

*“They have no choice; students lack options for specialized study in Nepal. Therefore, after completing their MBBS, we must provide quality educational choices here to retain them. Successfully mobilizing these professionals to stay in Nepal after five years of studies would be a significant achievement for us.”* — a representative from the MoHP.

By offering hands-on training, internships, and placement opportunities, universities can effectively prepare medical students for careers in specialized fields. These programs could encompass a variety of specialties, allowing healthcare professionals to advance their skills locally and thereby reducing the incentive to migrate abroad for further education.

## Discussion

The findings of this study highlight the challenges imposed by the brain drain of cardiac and other healthcare workers in the Nepalese health system. The analysis reveals that low pay scales, unsatisfactory working environments, aspirations for higher education, and concerns about quality of life are among the primary drivers leading skilled professionals to seek opportunities abroad. While the Nepalese government has implemented retention schemes for health providers, these initiatives predominantly target public sector workers, leaving those in the private sector without similar support mechanisms.

The consequences of brain drain are multifaceted, impacting Nepal’s healthcare system both positively and negatively. On the one hand, the loss of skilled and talented health manpower exacerbates existing shortages and strains healthcare delivery, especially in remote areas where the majority of sanctioned positions remain vacant. This mirrors similar challenges faced by other developing countries, such as Ghana and Kenya, where inadequate staffing levels impede the provision of essential healthcare services [21].

The ratio of cardiologists/patients is very low in Nepal which is 0.0057 per thousand population and the doctors-patient ratio is 1:1724 [22]. The nurse/patient ratio is 3.3 per thousand population [23]. The ratio is even poorer in African countries. The small country of Lesotho in southern Africa has 0.05 doctors per 1,000 population [21]. On the other hand, the US had an estimated ratio of 2.56 doctors per 1,000 population [21]. The discrepancy in healthcare workforce ratios between Nepal and developed countries like the United States and the United Kingdom highlights the stark contrast in working environments and facilities available to healthcare professionals. The allure of better opportunities abroad, including superior working conditions and access to advanced resources, further incentivizes migration among Nepalese healthcare workers. Furthermore, the disparity in physician-to-population ratios between Nepal and developed countries like the United Kingdom underscores the exodus of healthcare professionals from countries with limited resources to those offering better pay scales and working conditions. The attraction of higher salaries and improved facilities in urban areas leads to a concentration of healthcare workers in private and urban settings, exacerbating disparities in access to care between rural and urban populations.

A study conducted in Ghana also found that the majority of their clinics and hospitals were unable to provide the full range of expected services, due to a lack of personnel [24]. Also in Kenya, there are only 20 physicians per 100,000 people and about 50% of doctors are practicing abroad [25,26]. Whereas, the United Kingdom, similar to the US, has 2.8 doctors per 1,000 people [27]. It is found that in the UK, 300 doctors are from Nepal [28]. The government of Nepal has a clear organogram and structure for all health facility setups but the majority of the sanctioned posts are vacant, especially in remote areas of the country [13]. Despite the government’s efforts to establish clear organizational structures for health facilities, the majority of sanctioned positions remain vacant, resulting in an inability to provide comprehensive healthcare services to the population.

About 2000 doctors and more than 7000 nurses graduate annually in Nepal [9,29], the majority of them are concentrated in private and urban areas because of the availability of better facilities in urban areas [13]. Though super specialty public hospitals have satisfying pay scales for nurses, there still exists a very high staff turnover rate among nurses. The high turnover rate among nurses and the migration of super-specialized doctors to private centers further exacerbate the shortage of healthcare professionals in the public sector. Similar trends have been observed in other countries, such as South Africa and the Philippines, where healthcare graduates migrate to developed countries in the pursuit of better pay and opportunities [30,31]. A study from South Africa reported that half of their graduates emigrate to developed countries for better pay scales [30]. Similarly, the rate of Filipino nurses working in other countries for money is increasing by 5.32% annually creating a huge loss to the primary countries [31].

The most frequently reported pulling factors include better job opportunities, higher paying scales, and improved working environments, notably workplace safety and security, in developed countries. Conversely, the study identifies young age, positive family attitudes towards migration, and low levels of patriotism among healthcare professionals as common pushing factors. Physicians from African and Asian countries are particularly drawn to countries offering higher wages and where the density of medical doctors is relatively low [2]. This trend is echoed in findings from the World Health Report, which identifies a multitude of reasons for health worker migration from developing to richer countries, including lack of employment opportunities, poor health system management, heavy workloads, inadequate facilities, declining healthcare status, insufficient living conditions, and high levels of violence and crime [2]. Similar kind of study carried out in Nepal found that 52.22% of participants were not pleased with their job in Nepal [32]. It was observed that a significant proportion of participants expressed dissatisfaction with their jobs, with modern facilities, age, and self-interest also emerged as driving factors for migration among nursing staff. These findings highlight the multifaceted nature of healthcare worker migration and the need for comprehensive strategies to address the root causes of brain drain.

The initiatives undertaken by the Government of Nepal and the Nick Simons Institute to support retention and improve the performance of staff in rural public hospitals represent important steps towards addressing the healthcare workforce shortage in remote areas. By implementing programs specifically tailored to remote hospitals and allocating dedicated resources for health workers, the government is demonstrating a commitment to strengthening healthcare delivery in underserved regions [33]. The Government has allocated a separate line item for health workers in Nepal. The line item includes wages, salaries, and training for health workers [34]. One notable strategy employed by the government is the requirement for medical doctors to complete two years of service in remote areas before their medical degree is approved. This approach not only incentivizes healthcare professionals to work in rural areas but also ensures that medical education is tied to service in areas of greatest need. Similar strategies have been successfully implemented in other countries like Thailand, where scholarship recipients are required to return and work in contracted hospitals for a specified period [35].

Policymakers play a critical role in designing strategies to attract and retain medical professionals, particularly in areas with a high demand for healthcare services. By considering factors such as job satisfaction, salary, training opportunities, and social accountability, policymakers can develop targeted interventions that incentivize healthcare workers to remain in the country and serve in underserved areas. Additionally, the government must create opportunities and implement reinforcement measures to support retention efforts. It is also essential for the health workforce to actively explore opportunities and cultivate an interest in working within their own country, especially in rural and remote areas where healthcare access is limited. Universities can contribute to this effort by emphasizing social accountability and community engagement throughout the education and training of medical professionals, fostering a sense of duty towards serving marginalized populations and addressing healthcare disparities. Through collaborative efforts between policymakers, the government, the health workforce, and universities, sustainable solutions can be developed to ensure equitable access to quality healthcare services for all citizens.

This study has three major strengths**. First,** our sample is diverse as we included all the cardiac care-related stakeholders (policymakers, professional council representatives, representatives from organizations that advocate for patients, and public and private medical university deans) in this study. **Second**, this study is among the very few research on brain drain, exploring the various reasons for this problem in Nepal. And **third**, this paper used an exhaustive desk review as well as qualitative interviews as methodological approaches. It is not devoid of limitations. We conducted this study as a part of the National Need Assessment for CVDs in Nepal. The collection of data only from the central-level health workers is one of the main limitations of this study as the data do not represent the perception of all three levels of the health system.

The study holds several global public health implications. By exploring the factors contributing to brain drain among cardiac healthcare workers in Nepal, the study sheds light on less-studied issues surrounding skilled migration within the cardiac healthcare sector. This understanding is essential for policymakers and healthcare leaders worldwide, especially in developing countries grappling with similar challenges in retaining skilled professionals. The identification of contributing factors provides valuable insights for developing effective retention strategies not only in Nepal but also in other countries facing similar healthcare workforce shortages. The study also underscores the disparities in healthcare workforce distribution between developing and developed countries. This disparity has significant implications for global health equity, as shortages of skilled healthcare workers in low-resource settings can exacerbate health disparities and hinder efforts to achieve universal health coverage.

## Conclusion

Brain drain exists in every single field of the healthcare profession in Nepal. Different pulling and pushing factors are responsible for the brain drain like safety, job opportunities, and satisfaction in paying scale. The government of Nepal has developed some retention policies for healthcare workers, but strict implementations are needed to prevent draining the health workforce to developed countries. Respondents affirmed there is adequate production of healthcare workers in Nepal as evidenced by staggering numbers of registered healthcare professionals. However, their utilization is minimal which fuels brain drain out of the country.

The situation in Nepal reflects a broader global trend of talent migration from developing to developed countries, driven by disparities in economic opportunity, infrastructure, and quality of life. While brain drain cannot be completely eradicated, policymakers can take steps to mitigate its impact by implementing targeted retention schemes and improving job security within the country. This includes ensuring equitable access to training, resources, and professional development opportunities across both public and private sectors, as well as addressing systemic issues contributing to workforce shortages, such as understaffing and inadequate infrastructure in rural areas.

Investing in the development of healthcare infrastructure and implementing targeted recruitment and retention programs can help address disparities among Cardiac healthcare workers. Additionally, fostering international collaborations and partnerships can facilitate knowledge exchange and capacity-building initiatives, strengthening Nepal’s healthcare system and reducing its reliance on foreign-trained professionals. By addressing the root causes of brain drain and investing in the retention and development of its healthcare workforce, Nepal can work towards achieving sustainable healthcare delivery and equitable access to quality services for all its citizens. Thus, the government must deescalate this huge gap in production and consumption in the nation itself.

## Supporting information

S1 Data Dataset or codebook used in the analysis. (XLSX)

S1 Checklist Inclusivity in global research. (DOCX)
